# Flexible Strain-Sensitive Silicone-CNT Sensor for Human Motion Detection

**DOI:** 10.3390/bioengineering9010036

**Published:** 2022-01-13

**Authors:** Natalia A. Demidenko, Artem V. Kuksin, Victoria V. Molodykh, Evgeny S. Pyankov, Levan P. Ichkitidze, Victoria A. Zaborova, Alexandr A. Tsymbal, Svetlana A. Tkachenko, Hassan Shafaei, Ekaterina Diachkova, Alexander Yu. Gerasimenko

**Affiliations:** 1 Institute of Biomedical Systems, National Research University of Electronic Technology, 124498 Moscow, Russia; nix007@mail.ru (A.V.K.); molodykh1999@gmail.com (V.V.M.); zugusik@gmail.com (E.S.P.); ichkitidze@bms.zone (L.P.I.); gerasimenko@bms.zone (A.Y.G.); 2 Institute of Bionic Technologies and Engineering, I.M. Sechenov First Moscow State Medical University, 119991 Moscow, Russia; 3 Institute of Clinical Medicine, I.M. Sechenov First Moscow State Medical University, 119991 Moscow, Russia; zaborova.va@mipt.ru; 4 Sports Adaptology Laboratory, Moscow Institute of Physics and Technology, 141701 Dolgoprudny, Russia; 5 Department of Pathophysiology, I.M. Sechenov First Moscow State Medical University, 119991 Moscow, Russia; tsymbal_a_a@staff.sechenov.ru; 6 Department of Physical Rehabilitation Massage and Health-Improving Physical Culture, Russian State University of Physical Education, Sport, Youth and Tourism (SCOLIPE), 105122 Moscow, Russia; tkachenko.sa@rgufk.ru (S.A.T.); hassanshafai37@yahoo.com (H.S.); 7 Department of Oral Surgery, Borovskiy Institute of Dentistry, I.M. Sechenov First Moscow State Medical University, 119991 Moscow, Russia; secu2003@mail.ru

**Keywords:** nanocomposites, strain sensors, carbon nanotubes, laser radiation, CNT networks, flexible bioelectronics, health monitoring, wearable electronics

## Abstract

This article describes the manufacturing technology of biocompatible flexible strain-sensitive sensor based on Ecoflex silicone and multi-walled carbon nanotubes (MWCNT). The sensor demonstrates resistive behavior. Structural, electrical, and mechanical characteristics are compared. It is shown that laser radiation significantly reduces the resistance of the material. Through laser radiation, electrically conductive networks of MWCNT are formed in a silicone matrix. The developed sensor demonstrates highly sensitive characteristics: gauge factor at 100% elongation −4.9, gauge factor at 90° bending −0.9%/deg, stretchability up to 725%, tensile strength 0.7 MPa, modulus of elasticity at 100% 46 kPa, and the temperature coefficient of resistance in the range of 30–40 °C is −2 × 10^−3^. There is a linear sensor response (with 1 ms response time) with a low hysteresis of ≤3%. An electronic unit for reading and processing sensor signals based on the ATXMEGA8E5-AU microcontroller has been developed. The unit was set to operate the sensor in the range of electrical resistance 5–150 kOhm. The Bluetooth module made it possible to transfer the received data to a personal computer. Currently, in the field of wearable technologies and health monitoring, a vital need is the development of flexible sensors attached to the human body to track various indicators. By integrating the sensor with the joints of the human hand, effective movement sensing has been demonstrated.

## 1. Introduction

Flexible strain sensors are in high demand in many areas of technology, such as biomedicine and healthcare (blood flow pulsation sensors [1], respiration detection [2], limb movement monitoring [3], muscle signal studies [4], electronic skin [5], etc.) machines [6], soft robotics [7], interactive games [8], and virtual reality [9], as well as various industrial applications (for example, wind pressure control sensors [10], piezotronic strain sensors for transistors [11], etc.). The high interest in the development of flexible sensors is due to their (1) large elongation, which allow them to be used for registering deformation in both low and large ranges, and (2) softness, which leads to a potentially simpler integration with the human body, and also makes it possible to solve the problem of rigidity and bulkiness of structures. In the field of wearable electronics, resistive and capacitive strain and pressure sensors are currently the most widely used and actively studied [12]. Existing types of strain sensors, such as fiber Bragg grating (FBG) [13], triboelectric [14], and piezoelectric [15] strain sensors, usually cannot take slow or static deformation due to fast charge transfer. In addition, their practical implementation as wearable devices on the skin remains difficult due to the sophisticated measurement equipment required. On the other hand, resistive [16] and capacitive [17] sensors require simpler measuring equipment and demonstrate high flexibility and stretchability. In general, sensors of capacitive and resistive types, in which deformation is measured by changing the capacitance or electrical resistance of strain-sensitive materials, respectively, show similar characteristics in all parameters. However, the sensitivity of resistive sensors in comparison with capacitive ones with the same manufacturing technology remains higher and less susceptible to interference [18].

In general, strain sensors are made from functional materials integrated into flexible substrates. For resistive sensors, these materials include carbon (carbon nanotubes (CNT) [19], graphene [20], carbon black [21], etc.), metals (metal particles [22], nanowires [23], films [24], etc.), and various electrically conductive polymers [25] (polypyrrole (PPy), polyaniline (PANI), poly (3,4-ethylenedioxythiophene) polystyrene sulfonate (PEDOT: PSS)). Elastomers (rubber [26], polydimethylsiloxane (PDMS) [27], Ecoflex silicone [28], thermoplastic polyurethane elastomer (TPU) [29], etc.) are most often used as flexible supporting materials/substrates (flexible matrices), as well as other synthetic and natural polymers. Moreover, strain sensors based on conducting hydrogels have been developed, the sensitivity of which is caused by ionic or electronic conductivity [30]. However, due to the peculiarities of the phase state of hydrogels, the manufacture of strain sensors based on hydrogels with high extensibility or torsion remains a difficult task. 

Of all the types of electrically conductive additives, CNT attract the most attention. They have ultra-light weight, large aspect ratio, outstanding electrical conductivity, high tensile strength, and high chemical and thermal stability [31]. Moreover, their ability to form percolation networks capable of self-healing after applied deformation makes nanotubes ideal candidates for the development of highly efficient flexible sensors [32]. It is important to consider human skin compatibility developing flexible sensors. Therefore, a flexible matrix should not only be biocompatible, highly elastic, but also have a modulus of elasticity comparable to a modulus of human skin. Silicones, especially Ecoflex [33], meet these criteria perfectly. Ecoflex is an environmentally sustainable polymer due to its water resistance, is suitable for long-term applications, and its biocompatibility allows it to be used as a skin attachment device without any limitation, irritation, or discomfort.

However, the problem of integrating electrically conductive additives into a flexible matrix remains. Due to the difference in the rigidity of the components, there is a problem of their incompatibility, poor adhesion to each other and, as a consequence, the appearance of the slip effect, which complicates production and leads to deterioration of characteristics [34]. Currently, the following methods of fabricating strain sensors based on carbon and silicone are most often used: transfer of an array of nanotubes [35], screen printing [36], CNT deposition [37], and dry spinning [38]. However, manufacturing often requires sophisticated equipment or chemical solvents, making manufacturing expensive and unsafe. Recently, an inexpensive method for obtaining porous graphene using laser irradiation of a polyimide film was reported [39]. Such laser-induced graphene (LIG) has found application for the manufacture of strain sensors, for example, on cellulose materials [40]. As far as we know, this method is not currently used in the context of carbon nanotubes. Another serious problem is connecting the sensor to power supplies, as well as collecting and processing the received signals. Much of the work conducted in this area has focused on improving the performance of flexible sensors rather than integrating them with electronics. 

In this paper, we propose a simple and reliable method for manufacturing a strain-sensitive resistive sensors based on the Ecoflex-CNT-Ecoflex sandwich structure. Using laser structuring, we formed strong conductive CNT networks in a silicone matrix, which significantly improved the characteristics of the strain sensors. The inclusion of CNT in the polymers leads to the formation of nanocomposites with high electrical conductivity at a low concentration of CNT [41]. The use of the laser structuring method proposed in this research makes it possible to further improve the electrical characteristics. The proposed method will make it possible to use the uniqueness of the advantages of CNT at a low cost. In addition, accompanying electronics to easily integrate with the strain sensors to provide a fully wearable device for collecting human strain data have been developed. The work of a strain gauge attached to the joints of a human hand was demonstrated. A comparative analysis of the technology proposed in the study with previously developed sensors demonstrates the competitive characteristics of our strain sensors (Table 1). The measured and calculated parameters of the proposed strain sensors based on MWCNT/Ecoflex manufactured by the laser structuring method are described in Section 2 “Materials and Methods” and Section 3 “Results and Discussion”.

The article has the following structure. Section 2 presents the characteristics of the using components, describes the sensors proposed manufacturing technology, and presents test methods and the mathematical apparatus used for calculations. Section 3 includes a detailed description of the developed sensors and their characteristics based on the test results. This section also provides a discussion of the obtained results. Section 4 contains concluding remarks.

## 2. Materials and Methods

This section includes descriptions and specifications of the components used to make the sensors. It also outlines the proposed strain-sensitive material manufacturing technology using laser structuring and presents the manufacture of an electronic unit for reading and processing signals from a sensitive material, as well as presenting test methods, equipment, and formulas used to quantify electrical, sensitive, and mechanical characteristics of the developed sensor.

### 2.1. Components

The developed strain sensors consisted of material that is sensitive to deformations and a portable compact electronic unit for signal reading and processing.

The strain-sensitive material was a composite constructed of multi-walled carbon nanotubes (MWCNT) and a silicone elastomer. MWCNT (NanoTechCenter Ltd., Tambov, Russia) were produced by CVD synthesis and were quasi-one-dimensional, nanoscale, filamentary formations of polycrystalline graphite, predominantly cylindrical in shape with an inner channel in form of powder, with the following measurements: outer diameter 8–30 nm, inner diameter 5–15 nm, length ≥ 20 μm, specific surface area ≥ 270 m^2^/g, bulk density 0.025–0.06 g/cm^3^. A silicone elastomer on platinum Ecoflex 00-10 (Smooth-On Inc, Macungie, PA, USA) was used as a matrix. Silicone is a liquid of two components, where the first (A) is the base part, and the second (B) is the polymerization initiator. It has the following characteristics: dynamic viscosity of Ecoflex 00-10 in mixed state 140 Pa∙s, Young’s modulus at 100% elongation 0.06 MPa, Shore hardness 10 A, density 1.04 g/cm^3^, operating temperature range from 19 °C up to 232 °C. Carbon fiber electrodes were included in the finished strain-sensitive material.

The package of the electronic unit included: an electronic system and software for electrical signals processing, a signal receiver from a strain-sensitive material, batteries—4 AA batteries; it is possible to connect the unit to a computer via a USB cable. The block was based on a 12-bit ATXMEGA8E5-AU (Microchip Technology Inc., Chandler, AZ, USA) microcontroller. The body of electronic unit was made of ABS plastic.

### 2.2. Manufacturing of the Strain-Sensitive Material

**Figure 1:**
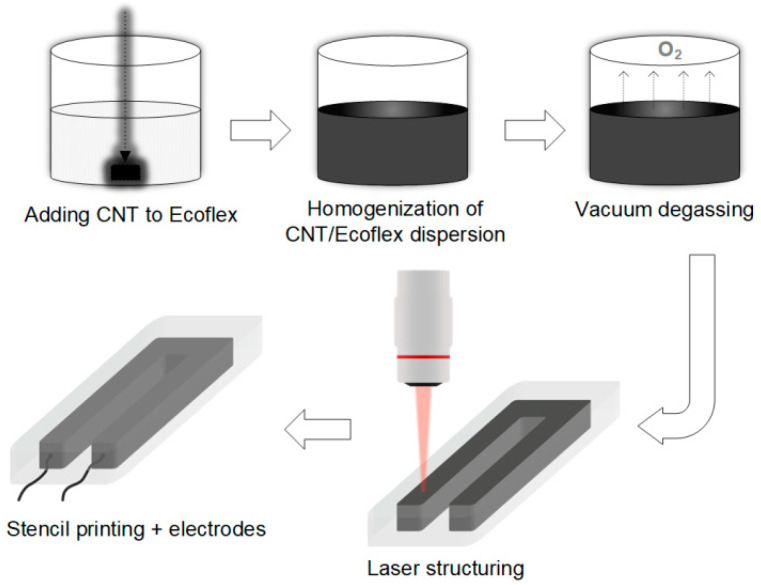
Strain-sensitive material manufacturing process.

A diagram of the strain-sensitive material manufacturing process is shown in Figure 1.

First, a 3D printer was used to print a mold from a photopolymer. In our case, the mold was rectangular, with the dimensions 3.5 × 1.5 × 2 mm, and had an internal U-shape hollowed section with dimensions of 3 × 1.2 × 1 mm. Next, a strain-sensitive material was manufactured, consisting of an Ecoflex/MWCNT composite. The first step was to add MWCNT to Ecoflex silicone in the liquid phase at a rate of MWCNT 3 wt.%. The resulting mixture of components was thoroughly mixed with a magnetic stirrer for at least 5 min for homogeneous distribution of nanotubes in silicone. To remove air microbubbles formed as a result of stirring, the mixture was placed in a vacuum chamber and the degassing process was started until the air bubbles were completely removed. Thereafter, the Ecoflex/MWCNT nanocomposite was prepared through the screen-printing method. For this, the Ecoflex/MWCNT mixture was placed in the inner U-shaped hollowed section of the mold. Next, the electrodes were added in a way that the mixture completely covered them. As a result, the electrodes had good adhesion to the material, which made it possible to effectively record the resistance values. The composite with electrodes in the mold was left at room temperature (23 ± 5 °C) until complete solidification was achieved (~4 h).

After complete polymerization, the resulting nanocomposite was subjected to laser structuring [46,47]. It was treated with laser radiation to reduce the resistivity values, form welded joints between nanotubes, and form a structured MWCNT conductive network inside the nanocomposite [48,49,50,51]. It is important to ensure the formation of contacts between nanotubes. The most crystalline contacts are formed when covalent C–C bonds appear [52]. During the formation of reliable contacts between nanotubes, the contact resistance decreases and, as a consequence, the electrical conductivity of the structures increases [53]. Moreover, it also increases the mechanical strength and fatigue strength of composites, which allows for the use of such materials for a long time [54]. After synthesis, nanotubes are mainly presented in the form of disordered systems [55], since the methods for synthesizing ordered nanoparticles are extremely difficult to control and difficult to implement. For this reason, methods for binding CNTs after synthesis by external influence are actively developing. Such methods are based on the mechanisms of action of concentrated energy, based on the latest advances in laser technology and precision mechanics. The use of the laser forming method proposed in this research makes it possible to further improve the electrical and mechanical characteristics [47,50]. The parameters of the laser irradiation were selected experimentally so as to prevent the combustion of the silicone. We used a pulsed Yb fiber laser with a wavelength of 1064 nm, radiation power was selected experimentally and amounted to 12 W, and irradiation time was 2 min. Finally, a laser-structured Ecoflex/MWCNT composite with electrodes was cast on both sides with layers of pure silicone to create an insulating and fully biocompatible coating. As a result of the simple manufacturing process, flexible and soft strain-sensitive material for the strain sensor was obtained, which is a sandwich structure: Ecoflex-CNT-Ecoflex.

In order to select the optimal concentration of MWCNT, rectangular Ecoflex/MWCNT composites with dimensions 3.5 × 1.5 × 1 and MWCNT concentrations 2, 3, 4 wt.% were initially manufactured. In order to assess the effect of laser radiation on electrical characteristics, the samples were subjected to laser structuring, as described above. Electrical resistance values were measured using UT33A+ (Uni-Trend Technology Co. Ltd., Dongguan, China) multimeter before and after laser structuring for each concentration of MWCNT.

Group of investigated sensors contained five samples from different batches to obtain statistical results during research.

### 2.3. Manufacturing of Electronic Circuit

**Figure 2:**
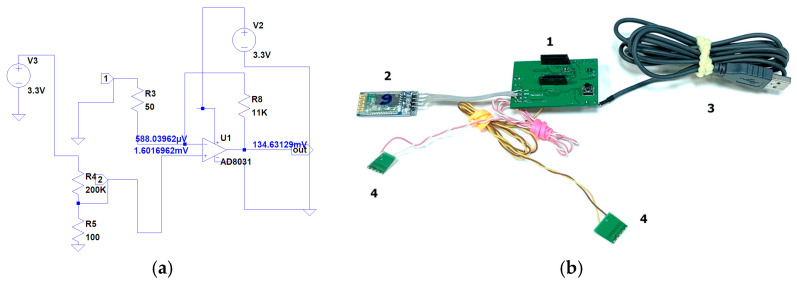
(**a**) Developed electronic circuit; (**b**) electronic microcontroller circuit layout, where 1—electronic system for processing electrical signals with a signal receiver from strain-sensitive material, 2—Bluetooth module for signal transmission to computer, 3—power cable, 4—wires for connecting of strain-sensitive material.

To create an electronic system for processing electrical signals (electronic unit) from a strain-sensitive material, an electrical circuit was developed using the LTspice software (Analog Devices Inc., Wilmington, NC, USA) (Figure 2a). Based on the resistance values of the material, the circuit was simulated, and values of resistors used in the circuit were selected. As a result of the experimental measurements, we determined the range of possible resistances of the strain sensors. A circuit converting resistance to voltage using an ATXMEGA8E5-AU microcontroller (Atmel Corporation, San Jose, CA, USA) equipped with an ADC (10 bit) was calculated. The input voltage range for this ADC was 0 to 1.14 V. The analog circuit was calculated so that the voltage converting the LM358D operational amplifier (STMicroelectronics, Geneva, Switzerland) from the sensor resistance was in the input voltage range. In this case, the sensor acts as a variable resistor. In the diagram, it is designated R4 (Figure 2a). In accordance with the developed circuit, an electronic microcontroller unit model was assembled from electronic components (Figure 2b). The electronic circuit (1) was based on the ATXMEGA8E5-AU microcontroller. The Bluetooth module (2) made it possible to transfer the data received from the strain-sensitive material to computer with installed software. Power was supplied by connecting the USB connector (3) to 5V power supply or to computer. Moreover, leads for connecting a strain-sensitive material were developed (4). Finally, a plastic body was made, in which the components of unit were placed, and in addition to the power cable, a block for batteries was added.

In this work, the electronic unit is designed specifically for our strain sensors, which have the specified characteristics. In the studies analyzed, commercial equipment is usually used to determine the electrophysical characteristics of sensors, and not a specially designed portable electronic unit. The electronic unit has a small size of 10 × 5 × 3 cm and a weight of 44 g and can be attached to clothing. The Bluetooth module and self-contained power module allow for data transfer to a computer without using additional wires. In our case, a complex device is presented: a sensor that includes flexible strain-sensor material and electronics. An experimental sample of a complex strain sensor device together with an electronic unit was developed according to the requirements of medical doctors from healthcare organizations. For example, in a study [56], the integration of a strain sensor with stretchable micro-supercapacitors is presented; however, a conventional multimeter is used to take the obtained values, as in most other studies.

### 2.4. Determination of Strain Sensor Sensitivity

**Figure 3:**
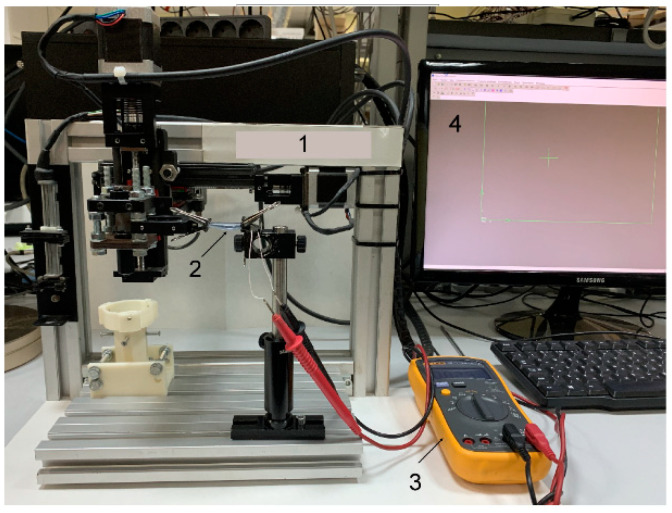
Installation for determining the sensitivity: 1—movement module with moving head in coordinates (x;y;z), 2—strain sensor, 3—digital multimeter connected to strain sensor, 4—personal computer for controlling the movement module.

To determine gauge factor (GF), studies of resistance on deformation dependence (tension/bending) were carried out using the installation (Figure 3). The installation consisted of a displacement module (1), containing a moving head, in which fasteners are provided for fixing the strain sensor (2), a multimeter (3), connected to strain sensor for recording electrical resistance values, and a personal computer (4) for controlling the displacement module. Movement trajectory was set using the movement module software. The strain-sensitive material was fixed in mountings of installation, the multimeter was connected to it, and the readings of electrical resistance were recorded with an extension every 5 mm.

Table 2 presents the meanings of symbols used in electrical and mechanical calculations.

Tensile GF was calculated by Formula (1):

GF_l_ = (ΔR/R_0_)/Ɛ. (1)

However, it is worth considering the influence of the Poisson ratio. When a strain sensor is stretched, it tends to contract in the transverse direction of stretching, depending on the Poisson’s ratio υ. According to Poisson’s law, the relationship between the deformation of cross section and the length can be calculated by Formula (2):

dA/A ≈ −2υƐ.(2)

The typically used Poisson ratio is υ = 0.495 [57]. In this case, the relative change in resistance can be written as (3):

ΔR/R_0_ = (1 + 2υ)Ɛ + Δp/p. (3)

Accordingly, when stretched, GF will take the form (4):

GF_lp_ = ((1 + 2υ)Ɛ + Δp/p)/Ɛ. (4)

The bending GF was calculated by Formula (5):

GF_θ_ = 100%·(ΔR/R_0_)/θ. (5)

The hysteresis was calculated by Formula (6):

h(%) = (R_t_ − R_c_)/(R_max_ − R_0_)·100%. (6)

### 2.5. Measurement of Strain Sensor Mechanical Characteristics

Using a digital multimeter Megeon 03100 (Megeon Llc., Zelenodolsk, Russia) and measuring ruler, applied force was determined depending on strain sensor elongation. Then, the elastic modulus was calculated using Formula (7):

*E* = F·l_0_/S·∆l. (7)

Maximum possible load (ultimate strength) that strain sensor can withstand was calculated using Formula (8):

σ = F_m_/A_0_. (8)

### 2.6. Investigation of Temperature Influence on Strain Sensor

The temperature coefficient of resistance was determined for temperature range 30–40 °C. To do this, strain sensors were placed on a heating table with temperature control function, and their resistance was recorded every 5 °C (from 25 to 50 °C). The temperature coefficient λ was calculated by Formula (9):

λ = ΔR/(R_0_·ΔT). (9)

### 2.7. Investigation of Strain Sensor Working Capacity

The working capacity of strain sensors was monitored at cyclic deformation. Strain sensors were attached to the finger joint using a polymer medical plaster to assess effectiveness in registering flexion-relaxing movements. In addition, a study to accurately determine the sensors response speed was carried out. Strain sensors were stretched by 50% and the time taken for the electrical resistance after deformation to return to its initial value was monitored.

## 3. Results and Discussion

This section includes a demonstration of the developed sensors, their structural, electrical, sensitive, mechanical characteristics, and performance under conditions of strain measurement, as well as a discussion of the results obtained.

### 3.1. Structure

**Figure 4:**
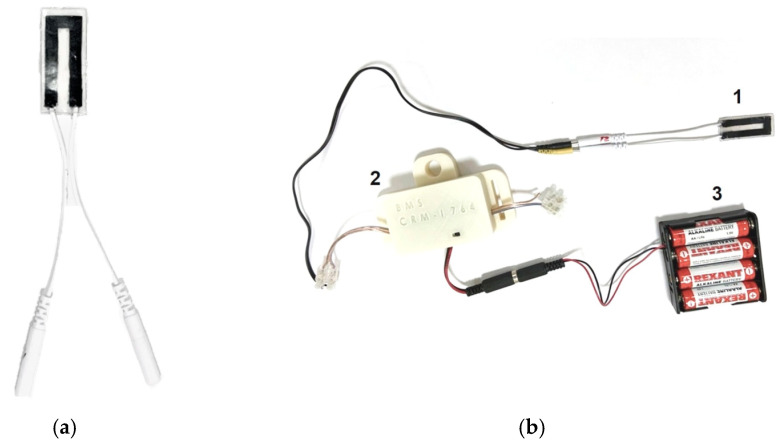
(**a**) Strain-sensitive material; (**b**) strain sensor, where 1—strain sensor, 2—electronic unit, 3—battery pack.

The appearance of the developed strain sensors is shown in Figure 4. The strain sensors consist of a strain-sensitive material, which is a flexible composite consisting of a biocompatible silicone elastomer and carbon nanotubes, equipped with electrodes (Figure 4a) and an electronic unit (Figure 4b).

**Figure 5:**
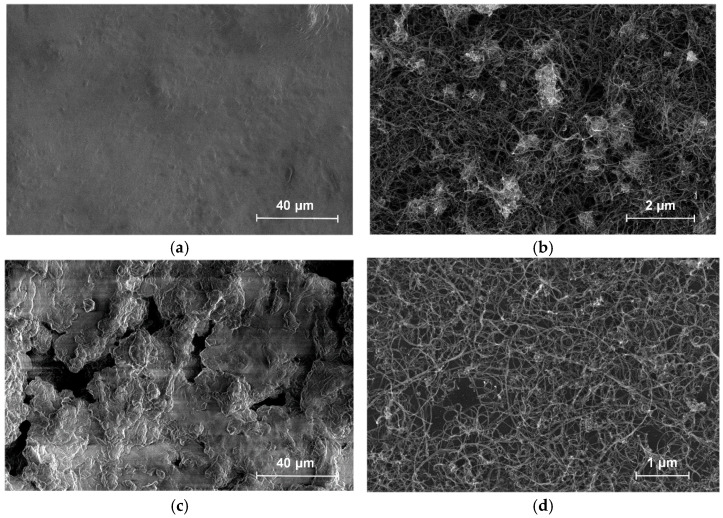
SEM of a strain-sensitive material without laser structuring: (**a**) Surface with ×2400 magnification; (**b**) internal structure of the MWCNT electrically conductive network with ×80,000 magnification. SEM of a strain-sensitive material after laser structuring; (**c**) surface with ×2400 magnification; (**d**) internal structure of the MWCNT electrically conductive network with ×60,000 magnification.

SEM images of the internal structure are shown in Figure 5. SEM images were obtained through scanning electron microscopy (SEM) using an FEI Helios NanoLab 650 microscope. The accelerating voltage of the electron column was 2 kV, and the current of the electron probe was 21 pA.

During the manufacturing process, the strain-sensitive material was irradiated with a pulsed laser, which left a characteristic deepening on the surface, as can be seen in Figure 5c. For comparison, Figure 5a shows the surface of the material without laser exposure. It can be seen that the silicone layer partially burnt out after laser structuring, which reduces the electrical resistance due to the change in the filler/matrix ratio. A network of carbon nanotubes is formed inside the material. Significant differences are noticeable in the appearance of the network without laser structuring (Figure 5b) and after laser structuring (Figure 5d). Laser radiation cleans the surface of nanotubes, and makes the network more uniform and rarefied. Rarefied conductive networks are known to achieve better strain sensitivity [58]. Nanotubes form long connections > 100 nm throughout the entire volume of the material (Figure 5d), providing functional characteristics and efficient operation of the strain sensor due to the high electrical conductivity and tenso-resistive properties of nanotubes [59].

### 3.2. Electrical Characteristics and Sensitivity

**Figure 6:**
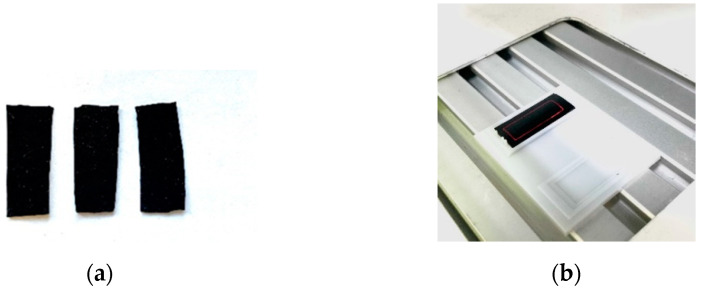
(**a**) Ecoflex/MWCNT composites with different concentration; (**b**) composite under a pilot laser beam.

The influence of the MWCNT concentration and laser structuring on the electrical resistance of the developed strain sensor was preliminarily estimated. Figure 6a shows a photo of the manufactured composites Ecoflex/MWCNT rectangular with dimensions of 3.5 × 1.5 × 1 cm and MWCNT concentrations of 2, 3, and 4 wt.%. Samples of each type were irradiated with a pulsed ytterbium fiber laser with a wavelength of 1064 nm and a power of 12 W, where the laser irradiation time was 2 min, to evaluate the effect of laser radiation on electrical resistance. Figure 6b shows a sample under a pilot laser beam. This percentage of nanotubes was selected taking into account the fact that lower percentages (2 wt.%) did not allow achieving sufficient electrical conductivity and, accordingly, sensitivity to deformations, especially at large deformations. A lot of electrically conductive filler is needed to keep the electrically conductive network stable. The higher percentage (4 wt.%) made fabrication more difficult, making the Ecoflex/MWCNT mixture highly viscous. In addition, according to the theory of percolation [60], high content of electrically conductive filler can impair sensitivity at small deformations.

Table 3 demonstrates that the resistance decreases with the increasing concentration of nanotubes. Composites structured by a laser are indicated in the table with the letter L. After laser exposure, the resistance of the samples decreased significantly (by several orders of magnitude).

Laser irradiation of polymer composites with CNT is one of the effective methods for modifying and improving the electrical characteristics of such composites [61]. In our case, the electrical resistance decreases for all concentrations of MWCNT. In this case, the samples were exposed to laser irradiation of the same power and duration. The decrease in resistance is most likely associated with the following factors. Laser irradiation causes pyrolysis of the silicone matrix, which leads to the formation of gaseous particles that leave the material, thereby increasing the concentration of MWCNT and decreasing the resistance of the material. Laser irradiation leads to modification of nanotubes due to thermal impact, reducing the content of impurities in CNT and defects. Nanotubes can be burned to amorphous carbon, which causes MWCNT to become rearranged in the silicone matrix. Amorphous carbon can act as solder and promote the formation of welded joints between individual nanotubes. All of these factors can contribute to the reduction in electrical resistance as a result of laser exposure.

Gauge factor for resistive sensors is defined as the relative change in electrical resistance to applied strain. Taking into account Poisson’s ratio, the average value is GF_lp_ ~3.6 (in the elongation range of 0–100%). However, it is worth noting that in the literature, Poisson’s ratio is often neglected and GF_l_ is indicated (that is, not taking into account Poisson ratio). In our case, the average GF_l_ ~4.9 (in the elongation range of 0–100%), with a maximum value of 7 (at 100% elongation). These values are higher than those of similar sensors based on Ecoflex and CNT silicone, for which the linear GF_l_ often does not exceed 2 [62]. Angular GF_θ_ was ~0.9%/deg (at bending 0–90°).

**Figure 7:**
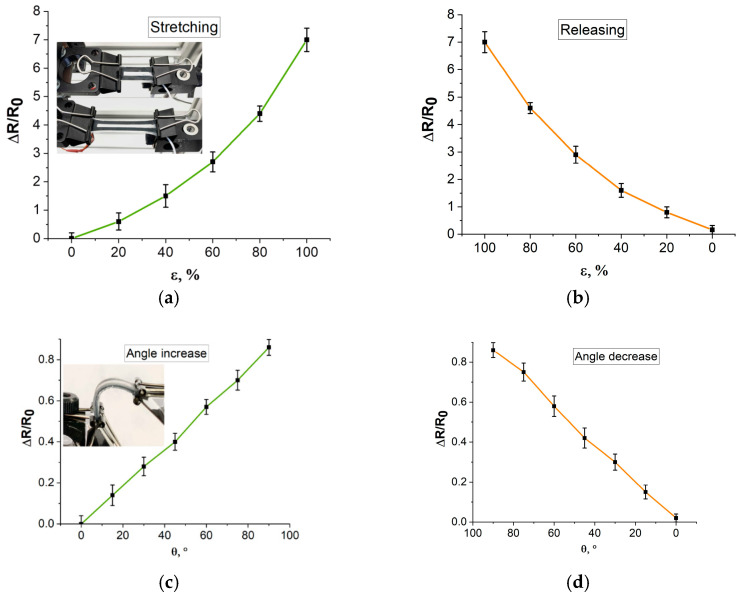
Resistance dependence of elongation while: (**a**) stretching (insert—the process of stretching the sensor), (**b**) releasing, resistance dependence of angle while; (**c**) increasing the angle (insert—the process of bending the sensor); (**d**) decreasing the angle.

Graphs of the dependence of the relative change in resistance from elongation and bending angle are shown in Figure 7. The graphs demonstrate the linear behavior of the strain sensor. The error in the figures is due to batch-to-batch variations of the sensor’s parameters.

It is known that a significant problem of strain sensors based on CNT and polymers is a high hysteresis, which leads to a nonlinear response under cyclic loads. Hysteresis is associated with a weak interfacial bond between hard CNT and soft polymers. The developed strain sensors demonstrate acceptable hysteresis values < 3% (2.3% for elongation, 2.6% for bending). Moreover, the high sensitivity to deformations allows the elongation/contraction or the increase/decrease in the bending angle to be clearly recorded, so that small fluctuations in resistance do not affect the performance of the strain sensors.

### 3.3. Mechanical Characteristics

The ability of strain sensor to withstand a load is directly related to its modulus of elasticity. In addition, for wearable, skin-mounted strain sensors, it is important that the modulus of elasticity is comparable to human skin so as not to cause discomfort. The average modulus of elasticity of the developed strain sensors at 100% elongation is 46 kPa, which is significantly less than the modulus of elasticity of the back of the skin. Therefore, for the dermis of the forearm and palm, the modulus of elasticity is in the range of 200 ± 50 to 250 ± 75 kPa [63].

**Figure 8:**
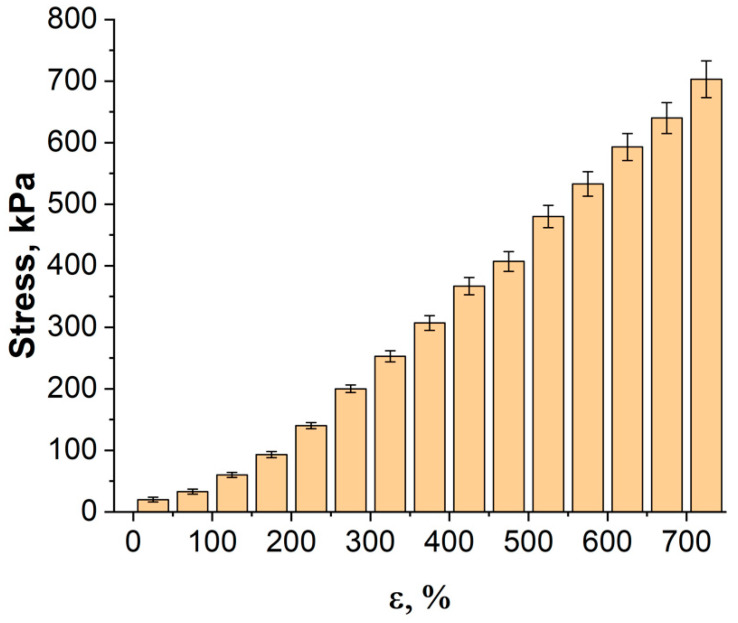
Diagram of applied tensile load of elongation of sensor.

When the applied force reaches a critical value-tensile strength, the strain sensor begins to collapse. The dependence of the applied load on the strain sensors elongation is shown in Figure 8. The average tensile strength was 0.7 MPa and corresponded to deformation of 725%.

The high tensile strength of the nanocomposite can be associated with excellent interfacial adhesion and load transfer between the CNT fillers and the Ecoflex matrix. Due to the strong bond between CNT and Ecoflex, slipping or delamination cannot occur between them. When high deformation is applied, the complex and entangled CNT network stretches and unfolds, but no break or fracture occurs due to the strong bond between the CNT and Ecoflex. Thus, the designed strain sensors can undergo very high deformation without causing electrical failure.

### 3.4. Resistance Dependence of Temperature

**Figure 9:**
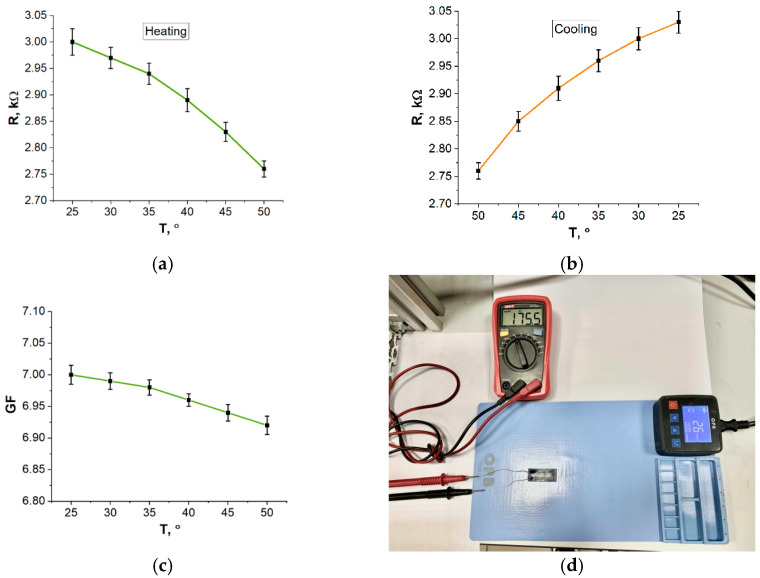
Temperature dependence of resistance (**a**) while heating, (**b**) while cooling, (**c**) GF dependence of temperature, (**d**) measurement of the change in resistance during heating.

Since the developed strain sensors are designed to work on the human body, it is necessary to take into account the effect of temperature on its performance, in particular, on electrical resistance. As the temperature rises, the strain sensor heats up, causing a decrease in resistance due to the generation of charge carriers included in the CNT (Figure 9).

In addition, CNT particles are encapsulated in a silicone matrix and, due to the thermal expansion of the matrix, the particles are compressed, leading to an increase in the contact area between the particles and the cross-section of the entire material, which also decreases the resistance [64]. However, the changes in resistance are negligible. For example, the temperature coefficient λ in the range of 30–40 °C ~ –2 × 10^−3^ testifies to the stability of the strain sensors installed on a human body.

### 3.5. Working Capacity

**Figure 10:**
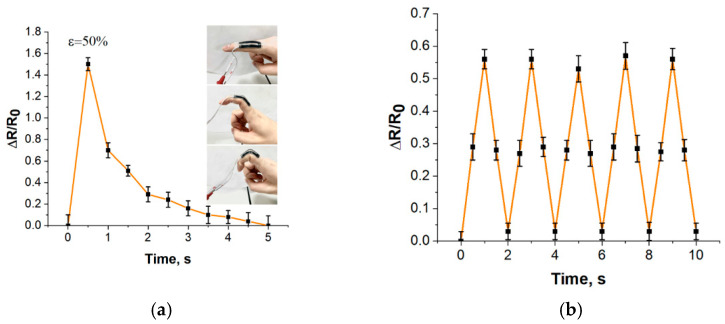
(**a**) Recovery time at 50% extension (insert—integration of the sensor with the finger joint); (**b**) response under cyclic loading during finger movement.

The study of the response rate of the strain sensors showed that after a deformation equal to 50%, the resistance of the strain sensors layer is fully restored and reaches its original value within 4 s (Figure 10a). It was found that the preliminary training of the strain sensor, which is a cyclic stretching of 10% of the length, allows the sensor to stabilize and respond to deformation with an instant recovery time up to 10% higher than the initial value [65]. In the current study, after the pre-training process, during cyclic deformation, the strain sensors stabilize to a resistance value equal to 103% of the initial one, and when the cycle value is repeated, this is achieved immediately after the termination of stretching (Figure 10b).

During cyclic changes in loading–unloading, some of the old bonds between nanotubes are deformed and not restored, but new bonds do appear. Therefore, at the initial stage of the cycle, hysteresis is recorded in the dependences of ΔR/R_0_ on ε, but after numerous load–unload cycles, i.e., after the so-called “training” process, hysteresis is minimized due to the establishment of a stable balance between “lost” and “restored” bonds between nanotubes.

The resulting strain sensors are thin and flexible, thus they can be fixed even on objects with complex geometric shapes. In the study, strain sensors were attached to a finger joint to track cyclic deformation. The sensors could reversibly track the slightest changes in finger position with a fast response time of 1 ms. A response delay exists in all polymer-based strain sensors due to the viscoelastic nature of the polymer.

The stability of the sensors was monitored for one month. The sensors were subjected to a daily cyclic tensile load of 50% of the original length and subsequent compression using the installation (Figure 3). The number of repetitions in the cycle was 100.

**Figure 11:**
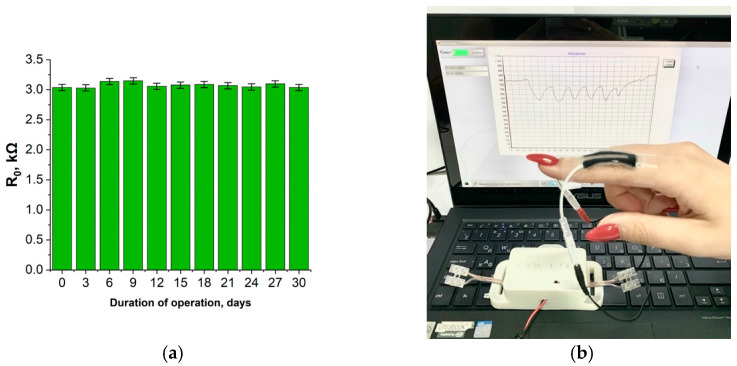
(**a**) Resistance value of the strain sensor after cyclic tests monitored for a month; (**b**) procedure for real-time measurement of finger joint movement.

The results of the study revealed that regular loads do not have a negative effect on the performance of the sensors. The initial resistance value remains constant using sensors for a month (Figure 11a).

Figure 11b shows the test process for measuring finger joint motion. The change in resistance of the strain sensor caused by flexion of the finger joint is transmitted to the computer utilizing an electronic unit. The developed software graphs the movement of the finger joint in real-time.

## 4. Conclusions

Currently, in the field of wearable technologies and health monitoring, an urgent demand is the development of flexible sensors that can be attached to the human body to track various indicators. This paper proposes a method for manufacturing a flexible strain gauge using laser formation of electrically conductive networks. The sensor shows linear resistive behavior and consists of a silicone elastomer and multi-walled carbon nanotubes. Carbon-based sensors typically show a high strain sensitivity range and excellent repeatability. However, due to strong π–π interactions, uniform dispersion of particles in a polymer matrix is problematic, thus it reduces the sensitivity of strain sensors. The proposed approach using laser structuring and the formation of electrically conductive networks of CNT inside a polymer silicone matrix can serve as an alternative to the existing methods of manufacturing and increasing the sensitivity of sensors with CNT. It is shown that laser radiation significantly reduces the resistance of the composite material MWCNT/silicone. The structural, electrical, and mechanical characteristics of the sensors are presented. The sensitive response of the sensors has been experimentally tested by integrating the sensors with a human arm joint. The sensors allowed tracking finger flexion/extension movements under cyclic load with a high sensitivity coefficient and low hysteresis. The obtained results demonstrate the great promise of the proposed approach for the implementation of multifunctional flexible sensors, especially in the field of orthopedics, where the measurement of joint movements during daily physical activity for long periods is of interest. The developed sensors demonstrate high elongation up to 725%, and remarkable sensitivity to deformations: gauge factor at 100% elongation −4.9, gauge factor at 90° bending −0.9%/deg, speed (response time 1 ms), and repeatability at cyclic load and integration with the human hand joint. The hysteresis of the developed sensors has a low value of ≤3%. The sensors demonstrate softness and elasticity: modulus of elasticity at 100% 46 kPa, high mechanical strength 0.7 MPa and thermal stability (the temperature coefficient of resistance in the range of 30–40 °C is −2 × 10^−3^).

In the future, further study of the sensor due to the large number of repetitions and the longer duration of both electrochemical and mechanical tests is planned, as well as a more detailed study of the sensitivity mechanism and the possibility of adjusting the output parameters of the sensor by changing the parameters of laser radiation (exposure time, power).

## Figures and Tables

**Table 1:** Comparison of the characteristics of the MWCNT/Ecoflex strain sensors with sensors manufactured by other methods.

Materials & Methods	ε, %	ΔR/R_0_	GF	σ, MPa	E, kPa	Response Time, ms
CNT/Ecoflex, Transfer of an Array of Nanotubes [42]	500	<10 (Depending on the Height of the CNT Array)	3–18	Not Identified	Not Identified	Not Identified
MWCNT/Ecoflex, CNT Deposition [43]	300	4 (ε = 80%); 0.45 θ = 90°	2.1	Not Identified	Not Identified	274
MWCNT/Ecoflex, CNT Deposition [37]	750	0.5 (ε = 100%)	0.65 (ε = 0–400%); 48 (ε = 400–700%)	Not Identified	Not Identified	Not Identified
MWCNT/Ecoflex, Screen Printing [36]	200	<0.5 (ε = 100%)	<0.4	0.82 ± 0.12	200	1.16
CNT/Rubber, Dry Spinning [44]	500	Not Identified	10.5	Not Identified	2000–5000	15
PSPI/PDMS, LIG [45]	125	Not Identified	380 (ε = 115–120%)	Not Identified	Not Identified	90
MWCNT/Ecoflex, Laser Structuring (This Work)	725	7 (ε = 100%)	7 (ε = 100%)	0.7	46	1

**Table 2:** Nomenclature table.

Symbol	Meaning
GF_l_	Tensile Gauge Factor
ΔR	Absolute Change in Resistance
R_0_	Initial Resistance Value
ΔR/R_0_	Relative Change in Resistance
Ɛ	Relative Change in Length
dA/A_0_	Relative Change in Cross-Sectional Area
υ	Poisson’s Ratio
Δp/p	Relative Change in Resistivity
GF_lp_	Stretching Gauge Factor
GF_θ_	Bending Gauge Factor
θ	Angle
h(%)	Hysteresis
R_t_	Tensile Resistance
R_c_	Compressive Resistance
R_max_	Maximum Resistance
E	Elastic Modulus
F	Applied Force
l_0_	Initial Sample Length
S	Surface Area to Which Force Was Applied
∆l	Elongation of Sample Due to Force Application
σ	Ultimate Strength
F_m_	Maximum Load Applied to the Sample
A_0_	Initial Cross-Sectional Area of Sample
λ	Temperature Coefficient
ΔT	Absolute Temperature Change

**Table 3:** Dependence of electrical resistance on the concentration of MWCNT.

MWCNT Concentration, wt.%	Resistance, Ohm
2%	500
3%	150
4%	100
2% + L	100
3% + L	40
4% + L	0.5

## Data Availability

The data presented in this study are available on request from the corresponding author.
